# Secretion of biologically active interferon-gamma inducible protein-10 (IP-10) by *Lactococcus lactis*

**DOI:** 10.1186/1475-2859-7-22

**Published:** 2008-07-28

**Authors:** Julio Villatoro-Hernandez, Maria J Loera-Arias, Anali Gamez-Escobedo, Moises Franco-Molina, Jorge G Gomez-Gutierrez, Humberto Rodriguez-Rocha, Yolanda Gutierrez-Puente, Odila Saucedo-Cardenas, Jesus Valdes-Flores, Roberto Montes-de-Oca-Luna

**Affiliations:** 1 Departamento de Histología, Facultad de Medicina, Universidad Autónoma de Nuevo León (UANL), Monterrey, N.L., México; 2 Departamento de Microbiología e Inmunología, Facultad de Ciencias Biológicas, UANL, San Nicolás de los Garza, N.L., México; 3 Departamento de Bioquímica, Facultad de Ciencias Biológicas, UANL, San Nicolás de los Garza, N.L,. México; 4 Departamento de Bioquímica, CINVESTAV, Apartado Postal 14-740, México; 5 División de Genética, Centro de Investigación Biomédica del Noreste, Instituto Mexicano del Seguro Social (IMSS), Monterrey, N.L., México

## Abstract

**Background:**

Chemokines are a large group of chemotactic cytokines that regulate and direct migration of leukocytes, activate inflammatory responses, and are involved in many other functions including regulation of tumor development. Interferon-gamma inducible-protein-10 (IP-10) is a member of the C-X-C subfamily of the chemokine family of cytokines. IP-10 specifically chemoattracts activated T lymphocytes, monocytes, and NK cells. IP-10 has been described also as a modulator of other antitumor cytokines. These properties make IP-10 a novel therapeutic molecule for the treatment of chronic and infectious diseases. Currently there are no suitable live biological systems to produce and secrete IP-10. *Lactococcus lactis *has been well-characterized over the years as a safe microorganism to produce heterologous proteins and to be used as a safe, live vaccine to deliver antigens and cytokines of interest. Here we report a recombinant strain of *L. lactis *genetically modified to produce and secrete biologically active IP-10.

**Results:**

The IP-10 coding region was isolated from human cDNA and cloned into an *L. lactis *expression plasmid under the regulation of the pNis promoter. By fusion to the usp45 secretion signal, IP-10 was addressed out of the cell. Western blot analysis demonstrated that recombinant strains of *L. lactis *secrete IP-10 into the culture medium. Neither degradation nor incomplete forms of IP-10 were detected in the cell or supernatant fractions of *L. lactis*. In addition, we demonstrated that the NICE (nisin-controlled gene expression) system was able to express IP-10 "de novo" even two hours after nisin removal. This human IP-10 protein secreted by *L. lactis *was biological active as demonstrated by Chemotaxis assay over human CD3+T lymphocytes.

**Conclusion:**

Expression and secretion of mature IP-10 was efficiently achieved by *L. lactis *forming an effective system to produce IP-10. This recombinant IP-10 is biologically active as demonstrated by its ability to chemoattract human CD3+ T lymphocytes. This strain of recombinant *L. lactis *represents a potentially useful tool to be used as a live vaccine *in vivo*.

## Background

Migration of immune cells at sites of antigenic challenge or lesions is mainly mediated by chemotactic cytokines called chemokines. Interferon-gamma inducible-protein-10 (IP-10) is a C-X-C cytokine that belongs to the subfamily of chemokines and is secreted by T cells, monocytes, endothelial cells, and keratinocytes [1,2]. IP-10 exerts a chemotactic effect on activated T lymphocytes and, Monocytes and NK cells [3,4]. It also has antitumor activity mediated by its angiostatic features, inhibiting tumor neovascularization and promoting damage in established tumor vasculature followed by necrosis *in vivo *[5-7]. Microbiological studies in radial diffusion assays revealed an antimicrobial activity of IP-10 against *Escherichia coli *and *Lysteria monocytogenes *[8]. Another report using a murine experimental model showed that bacterial clearance of the pneumonia-causal agent *Klebsiella pneumoniae *was associated with the presence of IP-10 [9]. Related reports demonstrated an important function of IP-10 in the infection resolution of the intracellular pathogen *Chlamydia trachomatis*, in which the absence of IP-10 led to the evolution of the infection originated by the causal agent [10]. For parasites, IP-10 exerted a protective response against *Leishmania amazonensis *in mice, not only reducing the prevalence of infection and the parasitic burden but also delaying and diminishing lesions caused by this intracellular parasite [11]. All these findings formed our interest to make use of this chemokine and investigate its effect on different chronic or infectious diseases. To accomplish this goal, several approaches have been developed for the construction of an adenovirus encoding for the mature moiety of the IP-10. Narvaiza et al. in 2000 reported that the use of an adenovirus encoding for IP-10 (Ad-IP-10) injected intratumorally reduced the tumor diameter, but when injected simultaneously with an adenovirus coding for the interleukin-12 (Ad-IL-12) the tumors disappeared completely in all the mice tested [12]. To date there were no suitable, safe strategies to produce and deliver IP-10 in a reliable, feasible manner in animal models. In recent years the use of *L. lactis*, a nonpathogenic, noninvasive lactic-acid bacterium has yielded optimistic and promising results as a biological vector to produce heterologous proteins of therapeutic interest [13-16]. This innocuous food-grade bacterium has been manipulated to express cytokines and antigens of therapeutic and medical interest with high efficiency and with safe and well-characterized expression systems. The administration of this *L. lactis *intranasally, resulted in the generation of protective immune responses against chronic and infectious diseases [14,17,18].

In our work we genetically manipulated the food-grade bacterium *L. lactis *to produce and secrete chemokine IP-10. This effort is impelled by the solid precedents that have demonstrated that *L. lactis *is a safe microorganism that could be used for the production of molecules of medical interest, intended to cause immune responses and be employed as a live vaccine.

## Results and Discussion

### Construction of an inducible system for IP-10 secretion by *Lactococcus lactis*

**Figure 1:**
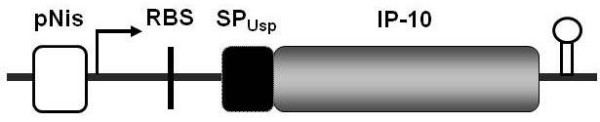
**Schematic design of IP-10 expression system for production and secretion by *Lactococcus lactis***. The scheme represents the final construction of the IP-10 inducible expression system carried by *Lactococcus lactis *(pSEC:huIP-10). The diagram shows the nisin-inducible promoter PnisA, the ribosome binding-site of *usp45 *(RBS), the usp45 signal peptide of the *usp45 *gene (SPusp), and the coding region for the mature moiety of IP-10. The open circle represents a rho-independent *trpA *transcription terminator fused just downstream to the IP-10 gene (not to scale).

To obtain a template to amplify the coding sequence for the mature IP-10, a cDNA was generated from total RNA isolated from human macrophages (see material and methods for more details). A 231-bp DNA fragment encoding IP-10 was amplified and cloned into the pCR 2.1 vector. This new plasmid was named pCR-hIP-10 and was transformed into *E. coli *DH5α. The identity of the human *ip-10 *gene was confirmed by sequencing. This construction was digested with *Nsi*I-*EcoR*I releasing the IP-10 coding region without ATG and then subcloned into the pVE:3684-E7 vector digested with the same enzymes. This IP-10 coding region was subcloned in-frame with the secretion-signal peptide-encoding sequence of the *usp45 *gene of *L. lactis *and under control of the nisin-inducing promoter Pnis [19]. This final construct, presented in Figure 1, was named plasmid pSEC:huIP-10 and was used to transform *L. lactis *NZ9000 by electroporation. Transformants of *L. lactis *were referred as the NZpSEC:huIP-10 strain. Table 1 contains strains and plasmids used and constructed in this work.

**Table 1:** Bacterial strains and plasmids.

**Strains**	**Genotype**		**Reference**
*E. coli *DH5α	Wild type, plasmid free		
*L. lactis *MG1363	Wild type, plasmid free		Gasson, 1983
*L. lactis *NZ9000	MG1363 (*nisRK *genes into chromosome), plasmid free		Kuipers et al., 1998
NZ(pSEC:huIP-10)	MG1363 (*nisRK *genes into chromosome), pSEC:huIP-10		This work

**Plasmids**	**Replicon**	**Plasmid characteristics**	**Reference**

pCR:TOPO	ori pUC	Ap^r^	Invitrogen
pCR:huIP-10	ori pUC	Ap^r^, DNA fragment encoding the IP-10 mature moiety	This work
pSEC:E7	pWV01	Cm^r^; gene expressed from P_nisA _encodes SP_Usp_-*E7 *precursor	Bermúdez-Humarán et al., 2002
pSEC:huIP-10	pWV01	Cm^r^; gene expressed from P_nisA _encodes SP_Usp_-*huIP-10 *precursor	This work

### Secretion analysis of IP-10 by *Lactococcus lactis*

**Figure 2:**
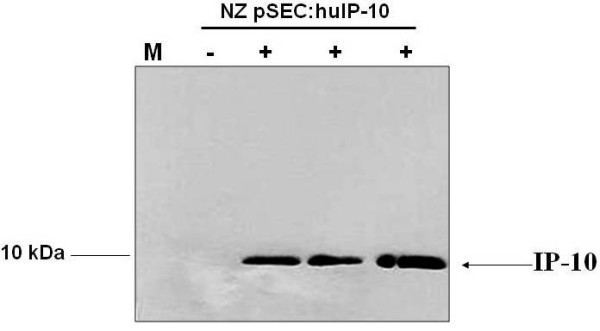
**Expression analysis of IP-10 secretion by recombinant *Lactococcus lactis***. Protein extracts from induced and noninduced cultures of recombinant *Lactococcus lactis *NZpSEC:huIP-10 were prepared from cell-free samples and analyzed by Western blotting using anti-IP-10. Mature IP-10 was detected in all the induced (+) cultures in the 10 kDa range as expected. No signal was found for noninduced cultures (-) of recombinant *L. lactis *NZpSEC:huIP-10. No immature or incomplete forms of IP-10 were detected. M, protein molecular marker.

The ability of strain NZpSEC:huIP-10 to produce and secrete IP-10 was examined by Western Blot using anti-human IP10 antibodies. Protein samples were prepared from cell (C) and supernatant (S) fractions of induced (+) and noninduced (-) NZpSEC:huIP-10 cultures. Supernatant fraction analysis from three recombinant-induced strains (+) showed a clear band corresponding to the size of the human IP-10 protein (Figure 2). No signal was detected either in the cell fraction of induced cultures (data not shown) or in the supernatant of noninduced recombinant strains (Figure 2). Different outcomes have been reported about the integrity of heterologous proteins produced by *L. lactis*. In most cases an efficient and correct expression was achieved, but sometimes a poor secretion or intracellular retention of incomplete proteins have been reported, even when this is directed by a secretion-signal peptide. We detected by Western blot a band corresponding to the size of the complete IP-10 protein in the supernatant fraction of induced cultures of *L. lactis *NZ pSEC:huIP-10. We did not find incomplete or immature forms of IP-10 in any fraction.

**Figure 3:**
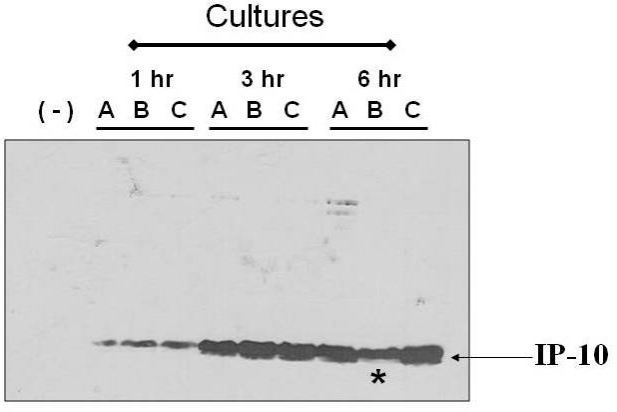
**De novo secreted IP-10 in the absence of nisin**. Three separate cultures (A, B, and C) of NZpSEC:huIP-10 were induced with 10 ng/ml of nisin for 1 hour. Culture A was in the presence of nisin all the time; culture B washed and suspended in fresh medium without inducer at hour 1 and 3, and culture C was washed and suspended in fresh medium without inducer only at hour 1. All cultures were grown a total of 6 hours. Protein extracts were analyzed by Western blot at hour 1, 3, and 6. The band in culture B at hour 6 (asterisk) represents the IP-10 "de novo" specifically secreted from hour 3 to 6 in the total absence of nisin demonstrating the ability of *Lactococcus lactis *to keep expressing and producing IP-10 for at least three hours.

Because we wanted to generate a lactococcal vehicle able to produce and secrete the human chemokine IP-10, to deliver IP-10 *in vivo*, we evaluated the ability of this recombinant strain to secrete IP-10 "de novo" over time after induction by nisin. To answer this, an experiment was made to specifically detect new IP-10 secretion after induction by nisin. Usually the time-course protein-expression experiments reported in the literature are done without removing the protein synthesized and accumulated over time, which may lead to misinterpretation. To avoid this we made the experiment shown in Figure 3. Three separate batches of recombinant *L. lactis *were induced with nisin (A, B, and C). One hour after nisin induction, cells in B and C were washed and suspended in new nisin-free medium (culture A was kept with nisin throughout the experiment). Two hours later cells in culture B were washed to remove the accumulated protein, suspended in new nisin-free medium and allowed to grow three more hours. A sample was taken for IP-10 detection by Western blot from the three cultures at different times, 1, 3, and 6 hours (counting time 0 as the time nisin was added). Results in Figure 3 show an IP-10 band of similar intensity in all cultures at one hour (A, B, and C) as expected because all of them were under the same conditions. This band corresponds to the amount of huIP-10 protein produced and accumulated during the first hour. Similar findings were observed at hour 3 where the intensity of the bands was equivalent in all three cultures. Because extracellular nisin had been removed from cultures B and C at one hour, their secreted IP-10 at hour 3 corresponds to the synthesized protein from the "switched on" operon induced by the intracellular nisin during the one-hour induction period. The secreted IP-10 detected at 6 hours in the supernatant of culture B represents the protein produced and secreted just in the last 3 hours, because washing the cells at hour 3 eliminated all the protein accumulated in the medium during the first three hours. This secreted IP-10 protein, although less than that in A and C at the same time point, corresponds to what we call "de novo" protein because secretion of this protein starts 2 hours after elimination of the nisin. At this point, we did not know if this last three hour IP-10 secretion in culture B is dependent on an intracellular-remaining inductor or translation of IP-10 mRNA still present in the cytoplasm.

Our time-correlated evaluation of IP-10 secretion at different times, removing all accumulated protein, avoids misinterpretations and allows pointing out the time that *L. lactis *is able to secrete recombinant proteins after inducing the activity of the pNis promoter. This experiment demonstrated that cultures of recombinant *L. lactis *continue expressing and secreting IP-10 for at least two hours after elimination of nisin from the culture media. For this reason, we would expect to find a similar secretion response from *L. lactis *after its administration into an animal model, where the inductor would no longer be available but where secretion efficiency would be influenced by the host environment.

### Recombinant IP-10 secreted by *Lactococcus lactis *is biologically active

Glycosylation is a common event caused by eukaryotic cells in a variety of proteins. This posttranslational modification has been seen to affect the activity of the protein itself. Because IP-10 has been reported as a glycoprotein [1] and because *L. lactis*, as other bacteria, does not cause glycosylation, we needed to determine if this *L. lactis *-secreted IP-10 was biologically active.

**Figure 4:**
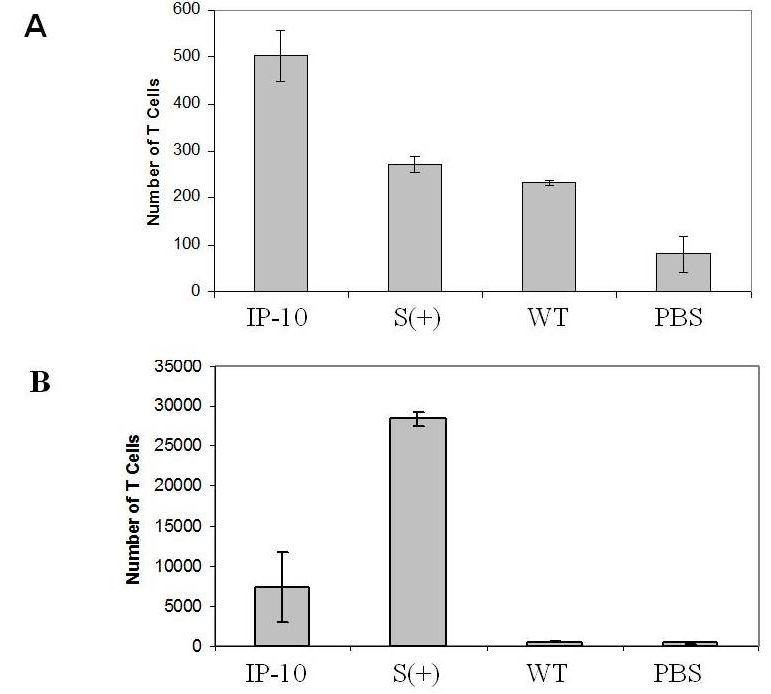
**Secreted IP-10 by *Lactococcus lactis *is biologically active**. Chemoattraction of *L. lactis*-secreted IP-10 was determined by using a Boyden chamber for chemotaxis. T lymphocytes obtained directly from human peripheral blood were stimulated with human IL-2 for 12 days. Chemotaxis assays using Boyden chambers were made with supernatants sterilized by filtration of both recombinant and wild-type *Lactococcus lactis*. To determine the number of chemoattracted cells, the membranes were stained with hematoxylin and the cells counted by using light microscopy at 1000 × (A). Cells in the lower chamber were incubated with specific anti-CD3+ and counted by FACS analysis (B). Supernatants of wild-type (WT) *L. lactis *and PBS were used as negative controls and Zymosan-activated serum (S+) as a positive control.

Chemoattraction of activated T lymphocytes is an important feature of IP-10. To evaluate the chemoattraction activity of the recombinant IP-10, human-peripheral T lymphocytes were activated with human IL-2 and subjected to an in vitro migration assay in a Boyden chamber (for more details see materials and methods). Cell-free supernatants from cultures of recombinant *L. lactis *were sterilized by filtration (0.22 μm) and added to the lower compartment of the Boyden chemotaxis chambers, with 100 000 IL-2 activated lymphocytes added to the upper wells. After 1 hour of chemotaxis and the counting of cells migrating to the lower side of the membrane (the one that was not exposed to the cells), we were able to demonstrate by direct observation that supernatants from cell-free cultures of recombinant strain NZ pSEC:huIP-10 significantly attracted more T lymphocytes than the controls *L. lactis *(Wild Type) and PBS, as demonstrated by the greater amount of cells on this underside (Fig 4A). Our conclusion is that presence of recombinant IP-10 in supernatants from induced NZ pSEC:huIP-10 cultures is able to interact specifically and chemoattract activated human T lymphocytes.

Chemotaxis activity on T lymphocytes was also evaluated by counting the cells that had migrated into the lower chamber by using a fluorescence-activated cell-sorting (FACS) flow cytometer using an anti-CD3 antibody (Fig 4B). The amount of cells detected by the flow cytometer in the positive control sample (Zymosan-activated human serum) showed the largest number of cells that did migrate all the way through the membrane, about 30 000 cells on average. Supernatants containing *L. lactis*-secreted IP-10 showed a significant chemoattraction to CD3+ human lymphocytes, counting > 5000 CD3+ T lymphocytes (Fig 4B). In contrast, few cells were chemoattracted to the lower chamber from the negative controls, PBS and supernatants from the wild-type *L. lactis*, as expected. These results effectively confirm that supernatants from recombinant *L. lactis *contain a biologically active human IP-10 secreted by this lactic acid bacterium.

## Conclusion

We developed a novel strain of *L. lactis *to secrete the antitumor chemokine IP-10 (Interferon-gamma inducible-protein-10). Its secretion was highly efficient because no immature or incomplete forms of this chemokine were detected in the cytoplasm or the media. Our results demonstrate that the recombinant strain NZ pSEC:huIP-10 of *L. lactis *produces and secretes biologically active human interferon-gamma inducible-protein-10 (IP-10) by using the nisin-controlled expression (NICE) system, as demonstrated by chemoattraction of human lymphocytes in a chemotaxis assay (Fig 4). We demonstrated that cultures of recombinant *L. lactis *once they are induced by nisin for only 1 hour actively secrete IP-10 protein "de novo" for more than two hours even when the inducer was removed. The chemoattraction ability of this recombinant hIP-10 plus its antitumor property makes this recombinant *L. lactis *strain expressing IP-10 a valuable tool for cancer therapy. Moreover, this strain of *L. lactis *able to secrete human IP-10 could be used as a mucosal enhancer to modulate or augment immune responses against tumors or infectious diseases.

## Methods

### Bacterial strains and growth conditions

Bacterial strains and plasmids used in this work are listed in Table 1. *Escherichia coli *DH5α was grown in Luria-Bertani (LB) broth at 37°C with vigorous agitation. *Lactococcus lactis *NZ9000 [20] was grown in M17 medium (DIFCO) supplemented with 1% glucose (GM17) at 30°C without agitation. Unless otherwise indicated, plasmid constructions were first established in *E. coli *and then transferred to *L. lactis *by electrotransformation as previously described [21]. Clones were selected by addition of 100 μg/ml of ampicillin or 10 μg/ml of chloramphenicol for *E. coli *and 10 μg/ml of chloramphenicol for *L. lactis*.

General procedures for DNA isolations and manipulations were made essentially as described [22]. PCR was done using *Vent *DNA Polymerase (New England Biolabs) and RT-PCR (HS RT-PCR, Sigma) as recommended by the manufacturer using the programmable thermal controller PTC-100 (MJ Research, Inc).

### Harvest and culture of macrophages

Human macrophages were isolated from donor whole blood by centrifugation on Histopaque-1077 (Sigma) according to the manufacturer. The layer containing mononuclear cells was carefully recovered, washed, and cultured for 3 hours in RPMI 1640 medium supplemented with 10% heat-inactivated fetal bovine serum (FBS), 1% penicillin-streptomycin solution, and 1% HEPES buffer and then incubated at 37°C in a 5% CO_2 _atmosphere. Macrophages were semipurified by removing nonadherent cells. Macrophages were seeded into 6-well-plates (Costar) at 5 × 10^6 ^cells/ml in complete RPMI-1640 medium (10% heat-inactivated FBS, 1% penicillin-streptomycin solution, and 1% HEPES buffer) stimulated with 20 mg/ml of lipopolysaccharides (LPS B from *Escherichia coli *026:B6, Sigma) and incubated at 37°C in a 5% CO_2 _atmosphere for 5 hours.

### Synthesis of Human cDNA

The total RNA from 5 × 10^6 ^cells of LPS-stimulated human macrophages (previously cultured and harvested) was isolated using TRizol reagent (Gibco) according to the manufacturer's instructions. The concentration and integrity of RNA was determined by measuring absorbance at 260 nm and analyzed by formaldehyde-agarose gel electrophoresis. The first strand cDNA was synthesized from 1 μg of total RNA by SuperscriptTM II reverse transcriptase (Gibco) and oligo (dT) 12–18 primer and used to synthesize the second strand.

### Inducible Expression of recombinant hIP-10 by *Lactococcus lactis*

To allow the expression of huIP-10, cultures of recombinant strains of *L. lactis *(OD_600 nm _= 0.6–0.8) were induced with 10 ng/ml of nisin (Sigma) for one hour.

For long-term expression experiments, three cultures of recombinant *L. lactis *were simultaneously induced with 10 ng/ml of nisin at OD_600 nm _= 0.4 and their growth was followed for 6 hours under optimum conditions. Culture A was left intact and no removal of medium was made at any time. Cultures B and C, after 1-hour induction, were centrifuged, the cell pellet washed with sterile PBS, and resuspended in the same volume of fresh GM17 medium. Culture B underwent the same wash step at hour 3. Protein extraction for all the 3 cultures was done identically at hour 1, 3, and 6.

### Protein extraction and Western Blotting

Cell and supernatant fractions were prepared separately. Samples were processed from 1.35 ml of culture. Cell pellets were obtained by centrifugation at 21000 × *g *at 4°C for 5 minutes and resuspended in 100 μl of TES-lysis buffer (25% sucrose, 1 mM EDTA, 50 mM TRIS·HCL, pH 8.0, lyzozyme [10 mg/ml] complemented with 1 mM phenylmethylsulfonylfluoride (PMSF) and 10 mM of dithiothreitol (DTT). The mixture was incubated at 37°C for 1 hour and then 50 μl of 20% SDS and one volume of loading buffer were added. The samples were maintained at -20°C before loading onto the gel.

The supernatant samples were treated with 1 mM of PMSF and 10 mM DTT to avoid proteolysis. Proteins were precipitated using 150 μl of 100% trichloroacetic acid (TCA) and incubated on ice for 10 minutes followed by centrifugation at 21000 × *g *at 4°C for 15 minutes. The pellet was resuspended in 50 μl of 50 mM NaOH and 50 μl of SDS-PAGE loading buffer (100 mM TRIS·HCl, pH 6.8, 200 mM dithiothreitol, 4% SDS, 0.1% bromophenol blue, and 10% glycerol). Twenty μl of these preparations were loaded onto 15% acrylamide gels. SDS-PAGE and Western blotting was done essentially as described [22]. Inmunodetection was done by the use of polyclonal anti-IP-10 (RnD Systems) as a primary antibody and protein-G horseradish-peroxidase conjugate (BioRad) and the SuperSignal West Pico Chemiluminiscent Substrate (Pierce) as recommended by the suppliers.

### Lymphocyte culture

Human-peripheral blood lymphocytes were isolated from donor whole blood by centrifugation on Histopaque-1077 (Sigma) according to manufacturer's instructions. The mononuclear cell layer was carefully recovered and washed three times with RPMI 1640 medium (Gibco). Adherent cells were discarded and cells in suspension were cultured in RPMI 1640 medium supplemented with 5% human serum (Sigma) and 200 U/ml human IL-2 (Santa Cruz Biotechnologies) at 37°C in a 5% CO_2 _atmosphere for 12 days. Cell density was kept all the time between 1 and 3 × 10^6 ^cells/ml.

### Chemotaxis assay and flow cytometry

Cell migration was measured in 5.0-μm pore-size cellulose-nitrate membranes (Whatman) using a Boyden chemotaxis chamber. Supernatants from recombinant and wild-type *L. lactis*, sterilized by filtration through a 0.22-μm filter (Millex, Millipore), were loaded into the lower compartment of the chamber. Lymphocytes (1 × 10^5 ^cells) in RPMI 1640 medium were loaded into the upper compartment of the chamber. Chemotaxis was allowed to occur for 1 hour at 37°C in 5% CO_2_. The membrane from the chamber was removed, washed with PBS, fixed, and stained with hematoxylin. Cells that had migrated to the underside of the filter were fixed with methanol and stained with hematoxylin. The number of migrated cells was counted by using light microscopy. For each membrane, five randomly selected fields were counted. Cells migrating into the lower compartment of the chamber were counted by a fluorescence-activated cell-sorting (FACS) flow cytometer (BD San Jose, CA). For this flow cytometry assay, the aqueous phase from the lower chambers was gently recovered and centrifuged. The resulting cell pellet was suspended in PBS and incubated for 10 min with human anti-CD3 FITC (BD Biosciences Pharmingen, San Diego, USA), washed with PBS, and counted with the FACS sorting cytometer. All chemotaxis assays were done in triplicate. Complement-activated serum was prepared from fresh human serum by addition of 25 mg of zymosan (Sigma) per ml of serum and incubated at 37°C for one hour. Statistical analysis was done using the Tukey test.

## Competing interests

The authors declare that they have no competing interests.

## Authors' contributions

JVH performed all experiments and drafted the manuscript. MJLA, AGE, MFM, JGGG and HRR participated in all experiments and technically supported the experimental process. YGP, OSC, JVF and RML contributed ideas for performing the experiments, professional support, and helpful suggestions for improving the manuscript. All authors read and approved the final manuscript.
